# Internet-Delivered Cognitive Behavioral Therapy for Anxiety Disorders in Open Community Versus Clinical Service Recruitment: Meta-Analysis

**DOI:** 10.2196/11706

**Published:** 2019-04-17

**Authors:** Geke Romijn, Neeltje Batelaan, Robin Kok, Jeroen Koning, Anton van Balkom, Nickolai Titov, Heleen Riper

**Affiliations:** 1 Department of Clinical, Neuro and Developmental Psychology VU University Amsterdam Public Health Research Institute Amsterdam Netherlands; 2 Altrecht Academic Anxiety Centre Utrecht Netherlands; 3 Department of Psychiatry VU University Medical Centre Amsterdam Public Health Research Institute Amsterdam Netherlands; 4 Department of Psychology University of Southern Denmark Odense Denmark; 5 Centre for Innovative Medical Technology Odense University Hospital Odense Denmark; 6 Pro Persona Psychiatric Centre Tiel Netherlands; 7 Mindspot Department of Psychology Macquarie University Sydney Australia; 8 Centre for Telepsychiatry Mental Health Services of Southern Denmark Odense Denmark

**Keywords:** anxiety disorders, cognitive behavioral therapy, internet, recruitment method, efficacy, effectiveness

## Abstract

**Background:**

Ample studies have shown the effectiveness of internet-delivered cognitive behavioral therapy (iCBT) for anxiety disorders. These studies recruited their participants mainly from the community and, to a lesser extent, from within routine care services. Little is known about whether different recruitment strategies lead to different treatment effects.

**Objective:**

This meta-analysis compared clinical results obtained in trials with recruitment from the community versus results obtained in trials with clinical service recruitment and explored factors that may mediate differences in treatment outcome.

**Methods:**

We included randomized controlled trials in which the clinical effects of iCBT for anxiety disorders were compared with a control condition (waitlist controls or face-to-face cognitive behavioral therapy). We classified trials as open recruitment trials (recruitment from the community) or clinical service recruitment trials (recruitment through outpatient clinics). Pooled effect sizes based on measures examining anxiety symptoms, depressive symptoms, and quality of life were computed for each type of trial. Subgroup analyses examined whether clinical results from open recruitment trials differed from those obtained in clinical service recruitment trials. Additional analyses explored which demographic, clinical, and treatment-related factors contributed to differences in effect sizes of open recruitment versus clinical service recruitment trials.

**Results:**

We included 42 studies with 53 comparisons (43 open recruitment comparisons and 10 clinical recruitment comparisons). Analyses of anxiety measures revealed, first, that iCBT open recruitment studies with waitlist control comparators showed a significantly higher effect size for decrease in anxiety symptoms than did those with clinical recruitment (Q=10.09; *P*=.001). This association between recruitment method and effect size was no longer significant in a multivariate metaregression with treatment adherence and exclusion of patients with depressive symptoms entered as additional predictors of effect size. Second, effect size for decrease in anxiety symptoms did not differ significantly between clinical recruitment and open recruitment studies with face-to-face cognitive behavioral therapy comparators. The effects of open recruitment trials and clinical recruitment trials did not differ significantly for the secondary outcomes, compared with face-to-face cognitive behavioral therapy and waitlist controls.

**Conclusions:**

iCBT was effective in samples recruited in clinical practice, but effect sizes were smaller than those found in trials with an open recruitment method for studies with waitlist control comparators. Hence, for patients with anxiety disorders in routine care, the impact of iCBT may not be as positive as for study participants recruited from the community. The difference between open recruitment trials and clinical service recruitment trials might be partly explained by patients’ greater therapy adherence in open recruitment trials and the stricter exclusion of patients with severe depressive symptoms in these studies. Since most trials in this meta-analysis applied an open recruitment method, more studies with routine care populations are needed to further validate these findings.

## Introduction

### Background

Internet-delivered cognitive behavioral therapy (iCBT) for anxiety disorders has been tested in ample randomized controlled trials and several meta-analyses. These studies show the potential of iCBT to reduce anxiety symptoms among patients and general populations, indicating that iCBT is effective when compared with a waitlist control (WLC), with effect sizes in the moderate to large range. Studies also suggest that it is as effective as face-to-face cognitive behavioral therapy (CBT) in improving symptoms of anxiety [1-5], although these studies are limited in number. Furthermore, iCBT may minimize treatment barriers such as high costs due to reduced time needed by therapists to provide therapy [6,7] and scalability.

The majority of trials on iCBT apply an open recruitment (OR) strategy, inviting individuals with anxiety symptoms from within the community to directly partake in the research study. These participants refer themselves to such a study. Often these studies apply strict inclusion and exclusion criteria; for example, they may exclude patients taking psychoactive medication, patients with comorbid disorders, or severely depressed patients [8,9]. Clinical service recruitment (CSR) trials, on the other hand, invite patients already seeking treatment in clinical practices to participate. Trials with an OR method provide evidence more related to *efficacy* (investigating whether a treatment works under ideal circumstances, with high internal validity), as opposed to CSR trials that are more related to *effectiveness* and provide information on whether a treatment works in clinically representative conditions [10].

Only a minority of the patient samples in trials with an OR method correspond to patient populations in a regular clinical setting in terms of sociodemographic characteristics, motivation for treatment, level of suffering, and clinical characteristics such as severity of anxiety, comorbidity, or medical history [11-13]. Furthermore, the use of extensive exclusion criteria in OR trials can reduce the degree to which these study samples resemble clinical populations in routine care settings. A meta-analysis [14] found a strong and positive relationship (*r*=.70) between the number of exclusion criteria and the rate of clinically improved participants for studies on iCBT for anxiety disorders. These results suggest a lower clinical effectiveness in clinically representative studies than in highly controlled studies. This raises the question whether results from OR trials can be extrapolated to routine clinical practice.

On the other hand, uncontrolled effectiveness studies show large clinical effects [15-20], thereby suggesting that iCBT for anxiety disorders may be as effective in routine care settings as demonstrated in efficacy trials. One review investigated controlled research of iCBT in routine clinical practice [21]. Results showed that effect sizes obtained from effectiveness studies (ranging from 0.75 to 1.73) were in the same range as those obtained in efficacy trials, though only 3 randomized controlled trials were included.

### Objective

Although several meta-analyses for (internet-based treatment of) anxiety disorders have been conducted in recent years, to our knowledge, none of these studies have compared the potential differences in clinical effectiveness between OR and CSR trials. In this study, we aimed to (1) assess whether OR trials produced clinical effectiveness for anxiety symptoms similar to that of CSR trials and (2) explore predictors of potential effect differences, such as demographic, clinical, and treatment-related characteristics. We based these predictors on differences between OR and CSR trials in patient samples and methods found in previous studies [11,13,14].

## Methods

### Study Retrieval

We report this meta-analysis in accordance with the Preferred Reporting Items for Systematic Reviews and Meta-Analyses (PRISMA) guidelines [22]. We retrieved studies through systematic literature searches in PubMed, PsycINFO, and EMBASE databases. Searches were conducted with keywords and text words, in which words indicative of internet treatment were combined with words indicative of anxiety disorder, CBT, and randomized controlled trial (see Multimedia Appendix 1 for the full search string). Furthermore, we checked reference lists of retrieved articles and of earlier reviews on iCBT for anxiety disorders [1-4].

### Inclusion and Exclusion Criteria

We included randomized controlled trials published up to and including December 2017 on guided and unguided iCBT and blended CBT for adults. Blended CBT combines face-to-face treatment with internet components into a single integrated treatment protocol [23]. We included only randomized controlled trials that assessed a primary diagnosis of an anxiety disorder according to the *Diagnostic and Statistical Manual of Mental Disorders* (Third Edition Revised [DSM-III-R], DSM (Fourth Edition [DSM-IV]), or DSM (Fifth Edition [DSM-5]) established by a structured diagnostic interview. We excluded studies on obsessive compulsive disorder [24] and posttraumatic stress disorder [25], since they are not classified as anxiety disorders in DSM-5. We included only randomized controlled trials published in English or Dutch.

#### Interventions and Comparators

We considered interventions to be CBT if they were based on cognitive behavioral principles [26] and consisted at least of cognitive restructuring or exposure (interoceptive exposure or exposure in vivo), or a combination of both. To be considered iCBT, the intervention must have been delivered (partly) via a computer or the internet through the use of webpages or email, or both. We included studies on iCBT targeting anxiety disorders and studies on transdiagnostic iCBT [27-31], addressing multiple anxiety disorders or addressing both anxiety and mood disorders, but only if participants had a diagnosis of an anxiety disorder and measures of anxiety were reported. We did not include interventions when the Web-based part of the treatment was limited to exposure scenes on a screen (eg, Heading et al [32]) because we considered this to be in virtuo exposure treatment, which is beyond the scope of this review.

iCBT was compared with WLC or regular face-to-face CBT treatment (including individual or group CBT delivered in a face-to-face format). We excluded studies with other comparisons such as transdiagnostic iCBT compared with disorder-specific iCBT, or guided iCBT versus self-help [24,25,33,34].

#### Outcome Measures

Our primary outcome was anxiety symptom severity based on the score on a rating scale used to measure general symptoms of anxiety. We applied a hierarchy of preferred outcomes for all measures based on frequency of use in the included trials. For general measures of anxiety, the preferred order was as follows: Beck Anxiety Inventory (BAI [35]), anxiety scale of the Depression Anxiety Stress Scales [36], State-Trait Anxiety Inventory [37], and Anxiety Sensitivity Index [38]. When a general measure of anxiety was not available, we used a measure for specific anxiety symptoms (see Multimedia Appendix 2 [39-49] for the order of rating scales and Multimedia Appendix 3 [7,27-31,50-85] for the outcome measures we used for all studies).

Since anxiety disorders are frequently accompanied by symptoms of depression and a reduced quality of life [86], secondary outcome measures were effects on depression severity and on quality of life (see Multimedia Appendix 2 for the order of rating scales [36,87-94]).

### Criteria for Open Recruitment and Clinical Service Recruitment

We classified trials as OR trials if participants were recruited from the community and referred themselves to be interested in the study in response to the invitation from a research team, by means of advertisements in newspapers or magazines, banners on websites, or large-scale mailings. In CSR trials, recruitment was carried out among patients already seeking treatment in outpatient clinical mental health practices. In the case of mixed recruitment strategies, we classified trials according to the most prominent recruitment strategy.

### Study Selection and Data Extraction

Two of the authors (GR and NB) independently screened the list of titles and abstracts that resulted from the literature search. Reference lists were screened for additional studies of relevance. We obtained full articles for potentially relevant abstracts according to the inclusion criteria. If included trials did not provide complete information, we contacted the primary investigator by email to attempt to obtain unreported data. We sent a second email when we received no response. Two researchers (GR and NB) extracted the data using Excel (2013) spreadsheets (Microsoft Corporation) and differences in such data were resolved by discussion. Extracted data (see Multimedia Appendix 3) included the study characteristics outlined in Textbox 1.

**Study characteristics extracted from the articles.**

- Year of publication
- Number of participants
- Recruitment setting (open or clinical service recruitment)
- Demographic characteristics of participants included in the study (sex, age, employment status [total rate of employed participants and rate of full-time employed participants], education level [rate of participants with college degree or higher])
- Anxiety severity at baseline
- Axis I comorbidity rate
- Exclusion criteria with regard to medication use (benzodiazepines and other psychoactive medication) and depressive symptoms (indicated by a score above a cutoff level on an outcome measure for depressive symptoms)
- Details of treatment conditions (duration and type of support provided by professionals)
- Outcome data
- Number of therapy sessions according to protocol, treatment dose (number of completed therapy sessions), and treatment adherence (number of completed sessions divided by the total number of sessions according to protocol)

### Risk-of-Bias Assessment

Two authors (GR and RK) independently assessed the risk of bias in the included studies based on 6 areas according to the Cochrane tool for assessing risk of bias [95]: (1) adequate generation of allocation sequence, (2) concealment of allocation to conditions (concealing allocation sequence from participants and investigators), (3) blinding of participants and personnel, (4) blinding of outcome assessors, (5) dealing with incomplete outcome data, and (6) selective outcome reporting (reported results give reason to suspect differences between reported and unreported findings). Because RK was an author of one of the included studies [50], this study was independently assessed by a third reviewer (NB). Discrepancies in scoring were resolved through discussion.

We assessed all areas as low, high, or unclear (ie, not enough information) risk of bias (see Multimedia Appendix 4). We assessed selective outcome reporting by comparing trial registrations with published articles, if available. When primary or secondary outcomes were missing, inserted, or changed in the article compared with the trial registration, or if secondary and primary outcomes had been switched, we deemed a study to be at high risk of selective outcome reporting. If no trial registration was available for a study, we coded the study as being at unknown risk for selective outcome reporting.

### Statistical Analyses

We used descriptive statistics to summarize demographic characteristics, and clinical and treatment-related characteristics of OR and CSR trials. We compared categorical variables using chi-square tests and continuous variables using *t* tests. We also compared the percentage of at-risk OR and CSR trials for all risk-of-bias indicators with chi-square tests.

We then calculated the pooled overall effect sizes (Hedges *g*) indicating the difference between the conditions at posttest and their 95% confidence intervals using the random-effects model with Comprehensive Meta-Analysis software version 3.0 (Biostat). Hedges *g* is an effect size that corrects for biases due to small sample sizes [96]. Effect sizes of 0.2, 0.5, and 0.8 indicate a small, moderate, and large effect size, respectively [97]. We used the effect sizes based on intent-to-treat analysis when available (in 51 comparisons); otherwise, we used complete-sample analysis results (in 2 comparisons [51,52]).

We examined heterogeneity among studies using Higgins I^2^ statistic. I^2^=0% reflects no heterogeneity; 25%, 50%, and 75% indicate a low, medium, and high level of heterogeneity, respectively [98]. A higher observed statistical heterogeneity indicates a higher proportion of observed variance, which can point to underlying differences between the pooled studies. This makes interpreting the pooled effect size difficult, as it is hard to distinguish the observed effect size from the true population effect size [99]. We also calculated 95% confidence intervals around I^2^ with the noncentral chi-square approach in the heterogi module for Stata 13.0SE (StataCorp LLC) [100].

We first calculated overall effect sizes on anxiety, depression, and quality-of-life treatment outcomes of iCBT compared with WLC and compared with face-to-face CBT. We then carried out subgroup analyses to assess whether clinical results from OR trials differed from those obtained in CSR trials. We performed these subgroup analyses according to the mixed-effects model, in which studies within subgroups are pooled with the random-effects model, and the fixed-effects model is used to test for significant differences among them by the between-subgroups Q-statistic [101].

We tested publication bias by inspecting the funnel plot and Egger test [102] on our primary outcome measure and by the Duval and Tweedie trim-and-fill procedure [103].

To more fully understand differences in effect size between OR and CSR trials, we conducted additional exploratory analyses. By means of subgroup and metaregression analyses, we examined which demographic, clinical, and treatment-related factors differed between OR and CSR trials and were associated with effect size on the primary outcome. Next, to examine whether these predictors contributed to the difference of effect size between OR and CSR trials, we tested recruitment method and the significant predictors in a multivariate model, except in case of collinearity. We assessed possible collinearity problems between predictors with the variance inflation factors. We considered variance inflation factor scores higher than 2.5 to indicate multicollinearity [104].

We also calculated the number needed to treat (NNT), according to Kraemer and Kupfer [105], and rounded upward to the next higher whole number [106]. The NNT gives some clinical context to statistical information, as it translates the magnitude of a statistical effect size into clinical implications—that is, the number of patients who must be treated to generate one more positive outcome than the same number of patients in the control group.

## Results

### Study Inclusion

**Figure 1:**
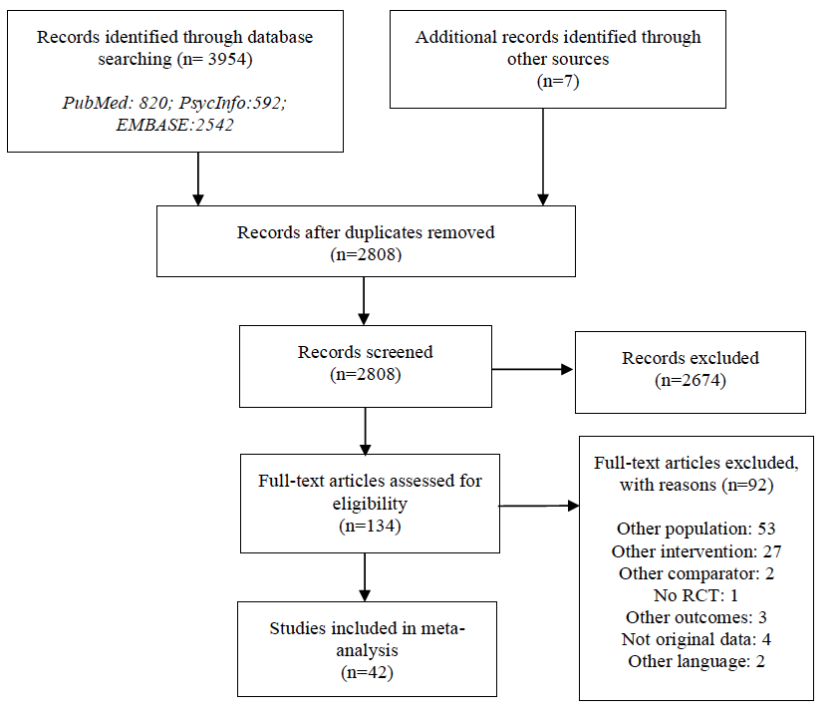
Preferred Reporting Items for Systematic Reviews and Meta-Analyses (PRISMA) flowchart of the selection and inclusion process. RCT: randomized controlled trial.

The literature searches retrieved a total of 3954 abstracts. Checking references of earlier reviews resulted in 7 more citations for consideration. After we removed duplicates, we screened 2808 abstracts. After screening abstracts, we retrieved 134 full-text articles for a more detailed evaluation of eligibility. Subsequently, we excluded 92 articles because they did not meet the inclusion criteria (Figure 1). We did not include 1 study because means and standard deviations for anxiety measures were not reported [107] and we received no response from the addressed researchers to our email questions regarding these issues.

### Description of Included Studies

We included a total of 42 trials (Multimedia Appendix 3). These trials entailed 53 comparisons of anxiety outcomes between iCBT and a control condition (WLC n=41, face-to-face CBT n=12) and included 3714 participants. A total of 45 comparisons entailed outcomes on depression and 21, on quality of life. Most studies were OR trials (31 trials with 41 comparisons), versus 8 CSR trials with 9 comparisons. In 3 studies both recruitment strategies were used [31,51,53]. In 2 of these studies, most participants were self-referred (n=129, 92.8% [31] and n=70, 77% [53]) and therefore we classified these studies as OR trials. In the third study, most participants were recruited through a clinical procedure (n=76, 67%) and therefore we classified this study as a CSR trial [51]. Thus, we classified a total of 33 trials with 43 comparisons as OR trials and 9 trials with 10 comparisons as CSR trials.

iCBT typically consisted of weekly sessions (ranging from 4 to 12 sessions for studies comparing iCBT with WLC and from 4 to 23 sessions for studies comparing iCBT with face-to-race CBT), with durations ranging from 4 weeks to longer than 3 months [54]. In 4 trials a self-help iCBT intervention (unguided) was offered; in the other trials iCBT was guided, meaning that online professional support was provided.

Table 1 displays demographic, clinical, and treatment-related characteristics of OR and CSR trials. We found significant differences between OR trials and CSR trials with regard to age of participants, sex, baseline severity, exclusion of severely depressed patients, treatment dose, and treatment adherence. We could compare baseline severity only for scores based on the BAI and the Social Phobia Scale, since these were the only outcome measures that were reported in both OR trials and CSR trials. We did not classify 3 OR trials [55-57] and 1 CSR trial [51] that mentioned exclusion of patients with depressive symptoms as such, because no definition in terms of a score on a measurement for depressive symptoms was provided.

**Table 1:** Comparison of demographic, clinical, and treatment-related characteristics of included open recruitment studies and clinical service recruitment studies^a^.

Characteristics			Open recruitment	Clinical recruitment	Test statistic	*P* value
**Demographic characteristics**						
	Age (years), mean (SD)		37.5 (5.4)	34.1 (1.7)	*t*_3657_=29.1	<.001
	Female sex, n (%)		1871 (67)	541 (58.7)	χ^2^_1_=21.2	<.001
	Education: college degree, n (%)		670 (53.4)	171 (49.4)	χ^2^_1_=1.7	.19
	Employed: full-time or part-time, n (%)		390 (58.7)	279 (59.7)	χ^2^_1_=0.1	.71
**Clinical characteristics**						
	**Primary diagnosis: trials that applied this diagnosis as inclusion criterion, n (%)**				χ^2^_4_=4.3	.37
		Panic disorder	13 (30.2)	3 (30)		
		Social anxiety disorder	14 (32.6)	3 (30)		
		Generalized anxiety disorder	7 (16.3)	0 (0)		
		Specific phobia	2 (4.7)	0 (0)		
		Multiple anxiety disorders	7 (16.3)	4 (40)		
	Baseline Beck Anxiety Inventory score, mean (SD)		25.4 (11.8)	29.0 (11.1)	*t*_1208_=–6.2	<.001
	Baseline Social Phobia Scale score, mean (SD)		36.0 (1.7)	43.0 (0.4)	*t*_606_=–82.7	<.001
	Comorbidity: comorbid Axis I diagnosis, %		57.6	58.1	χ^2^_1_=0.0	.87
	Exclusion benzodiazepines: trials that applied this criterion, n (%)		10 (23.3)	0 (0)	χ^2^_1_=2.2	.14
	Exclusion psychoactive medication other than benzodiazepines: trials that applied this criterion, n (%)		0 (0)	0 (0)	N/A^b^	N/A
	Exclusion severe depression: trials that applied this criterion, n (%)		25 (58.1)	2 (20)	χ^2^_1_=4.7	.03
**Treatment-related characteristics**						
	Treatment dose: number of completed sessions, mean (SD)		6.1 (1.4)	5.1 (2.4)	*t*_835_=9.45	<.001
	Treatment adherence: treatment completed %		77.9	53.1	*t*_1599_=44.73	<.001

^a^Means and percentages are based on studies these data were available for. All available data are reported in Multimedia Appendix 3.

^b^N/A: not applicable.

No other variables differed (see Table 1). None of the included studies excluded patients who used psychoactive medication other than benzodiazepines, although a stable dose for the past 1 to 3 months was a criterion for inclusion in general.

### Risk-of-Bias Assessment

For both OR trials and CSR trials, most of the studies scored a low risk on sequence allocation (OR: 27/33, 82%; CSR: 8/9, 89%), blinding of outcome assessors (OR: 33/33, 100%; CSR: 8/9, 89%), and completeness of outcome data (OR: 27/33, 82%; CSR: 6/9, 67%) (see Multimedia Appendix 4). On allocation concealment most CSR trials scored a low risk (7/9, 78%) compared with 12 of 33 (36%) OR trials. Only 4 of 33 (12%) OR and 2 of 9 (22%) CSR trials scored a low risk on selective outcome reporting, A total of 10 (30%) OR trials and 5 (56%) CSR trials scored a high risk because preregistered outcome measurements were not reported, or other outcome measurements that were not preregistered were inserted in the article. Additionally, 19 (58%) OR trials and 2 (22%) CSR trials were not registered in a trial database and we therefore scored them as having an unclear risk. We rated all included studies as having a high risk of bias on blinding of participants and personnel, because it is not possible to blind participants or therapists to the characteristics of the treatment that is offered.

We found no significant difference between the percentage of OR trials and the percentage of CSR trials with a high risk for any of the risk-of-bias indicators (*P* values ranged from *P=*.08 for sequence generation to *P*=.49 for allocation concealment).

### Overall Effect

#### Primary Outcome

The overall mean between-groups effect size of iCBT on anxiety symptom reduction when compared with WLC at posttest was *g*=0.72 (95% CI 0.60-0.83; *P*<.001) with moderate heterogeneity of I²=53% (95% CI 31-66) and NNT=3.

The difference in overall effect size for the decrease in anxiety symptoms between iCBT and face-to-face CBT at posttest was nonsignificant (*g*=0.12, 95% CI –0.02 to 0.26; *P*=.11; I²=0%, 95% CI 0-75; NNT=15).

#### Secondary Outcomes

Effect sizes of iCBT compared with WLC on depressive symptoms (*g*=0.61, 95% CI 0.46-0.75; *P*<.001; I²=70%, 95% CI 57-78; NNT=3) and quality-of-life measurements (*g*=0.44, 95% CI 0.33-0.55; *P*<.001; I²=5%, 95% CI 0-54; NNT=5) were moderate.

For iCBT compared with face-to-face CBT, effect sizes on depression measurements (*g*=0.04, 95% CI –0.13 to 0.21; *P*=.65; I²=19%. 95% CI 0-61; NNT=45) and quality-of-life outcomes (*g*=0.18, 95% CI –0.05 to 0.41; *P*=.12; I²=0%, 95% CI 0-85; NNT=10) were both nonsignificant.

### Open Recruitment Versus Clinical Service Recruitment

#### Primary Outcome

**Figure 2:**
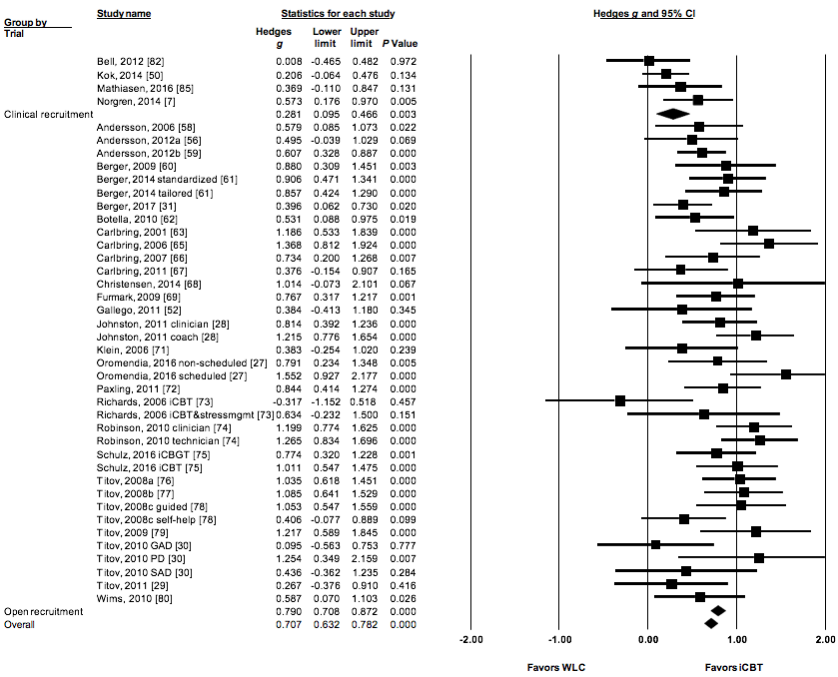
Forest plot of effects on anxiety symptoms of open recruitment trials and clinical service recruitment trials comparing internet-delivered cognitive behavioral therapy (iCBT) with waitlist control (WLC). GAD: generalized anxiety disorder; iCBGT: clinician-guided group iCBT; PD: panic disorder; SAD: social anxiety disorder.

For studies with WLC comparators, we found a significant difference between OR and CSR trials) in favor of OR trials (Q=10.09; *P*=.001) (Table 2 and Figure 2). The effect size on anxiety symptom reduction for OR trials was significant and large (*g*=0.79; *P*<.001) in favor of iCBT, whereas CSR trials obtained a small effect size (*g*=0.28; *P*=.003) in favor of iCBT.

**Figure 3:**
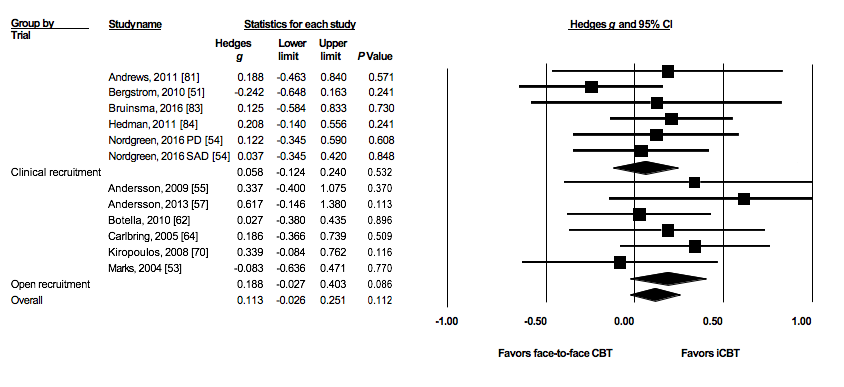
Forest plot of effects on anxiety symptoms of open recruitment trials and clinical service recruitment trials comparing internet-delivered cognitive behavioral therapy (iCBT) with face-to-face cognitive behavioral therapy (CBT). PD: panic disorder; SAD: social anxiety disorder.

We found no difference in anxiety symptom reduction between OR (n=6) and CSR trials (n=6) comparing iCBT with face-to-face CBT (Q=0.82; *P*=.37*)* (Table 2 and Figure 3). Both OR trials (*g*=0.19; *P*=.09) and CSR trials (*g*=0.06; *P*=.51) reported a nonsignificant difference between iCBT and face-to-face CBT on decrease in anxiety symptoms.

#### Secondary Outcomes

With regard to depressive symptoms, we found no significant difference between OR trials and CSR trials with WLC comparators (Q=1.43; *P*=.23) or face-to-face comparators (Q=0.85; *P*=.36).

For quality-of-life measurements, we found no significant difference between OR trials and CSR trials for studies comparing iCBT with WLC (Q=0.05; *P*=.83) or for studies comparing iCBT with face-to-face CBT (Q=0.48; *P*=.49).

Multimedia Appendix 5 presents a complete overview of results of OR and CSR subgroup analyses of secondary outcomes.

### Publication Bias

Neither visual inspection of the funnel plots (see Multimedia Appendix 6) and Egger test (WLC studies: intercept=0.83; 95% CI –0.90 to 2.56; *P*=.34; face-to-face CBT studies: intercept=1.12, 95% CI –0.93 to 3.17; *P*=.25) nor the Duval and Tweedie trim-and-fill procedure showed evidence of publication bias.

### Additional Exploratory Analyses

As Table 1 shows, several demographic (age, sex), clinical (baseline severity, exclusion of severely depressive patients), and treatment-related (treatment dose, treatment adherence) variables differed significantly between OR trials and CSR trials. Of these variables, only exclusion of severely depressed patients (Q=8.06; *P*=.005), treatment dose (slope=0.10; *P*=.003), and treatment adherence (slope=0.01; *P*<.001) appeared to be significantly associated with effect size for WLC comparators in separate subgroup (exclusion of severely depressed patients) and metaregression (treatment dose, treatment adherence) analyses (see Multimedia Appendix 7), meaning that the effect size was higher when severely depressed patients were excluded, when the treatment dose was higher, and when the adherence rate was higher.

In a multivariate analysis we explored whether the association between recruitment method and effect size for studies with WLC comparators was mediated by these variables. As the variance inflation factors between treatment dose and treatment adherence was 2.7, and treatment adherence was more significantly associated with effect size than treatment dose, we did not include treatment dose in the multivariate model.

Results showed that recruitment type (slope=0.30; *P*=.14) was no longer significantly associated with the effect size in the multivariate metaregression analysis, nor was treatment adherence (slope=0.01; *P*=.23) or exclusion of severely depressed patients (slope=0.13; *P*=.27).

**Table 2:** Main effects of open recruitment trials and clinical service recruitment trials comparing internet-delivered cognitive behavioral therapy versus waitlist control and versus face-to-face cognitive behavioral therapy at posttest, primary outcome.

Group		Comparisons, n	Respondents, n	Hedges *g* (95% CI)	*P* value	I^2^ (95% CI)	NNT^a^	Between-groups Q (*P* value)
**Waitlist control**								10.09 (.001)
	Open recruitment	37	2474	0.79 (0.71 to 0.87)	<.001	44 (6 to 58)	3	
	Clinical recruitment	4	446	0.28 (0.10 to 0.47)	.003	20 (0 to 85)	7	
**Face-to-face cognitive behavioral therapy**								0.82 (.37)
	Open recruitment	6	336	0.19 (–0.03 to 0.40)	.09	0 (0 to 75)	10	
	Clinical recruitment	6	452	0.06 (–0.12 to 0.24)	.53	0 (0 to 75)	30	

^a^NNT: number needed to treat.

## Discussion

### Principal Findings

This meta-analysis showed that iCBT is more effective than WLC in reducing anxiety symptoms at posttreatment. We found no indication for differences in effect sizes between iCBT and face-to-face CBT. These outcomes confirm the results of previous meta-analyses, which found moderate to large effect sizes for WLC comparator studies and small and nonsignificant effect sizes when comparing iCBT versus face-to-face CBT [1-4].

Our main research question was whether OR trials produce effects for anxiety symptoms similar to those of CSR trials. For studies with WLC comparators, recruitment method was significantly associated with anxiety treatment outcomes (Q=10.09; *P=*.001), indicating that effect sizes are higher in OR trials than in CSR trials.

We explored whether differences between characteristics of samples in OR trials and CSR trials might explain the gap between effects we found in favor of OR trials. Multivariate metaregression analysis revealed that the association between recruitment method and effect size may be partly explained by greater treatment adherence and the exclusion of severely depressed patients in OR trials.

For studies with face-to-face CBT comparators, we observed no difference in anxiety outcomes between OR trials and CSR trials (Q=0.82; *P*=.37). Possibly, the number of studies with face-to-face CBT comparators was too low, making these analyses underpowered to detect differences. Another explanation could be that studies with face-to-face CBT comparators resembled each other more on other criteria for clinical representativeness than studies with WLC comparators, as these studies are conducted in routine care. For example, in studies with face-to-face comparators, treatments are generally delivered by skilled clinicians and in clinically representative settings. These study characteristics are more varied in studies with WLC comparators, where treatments can also be delivered by researchers or graduate students and in a research setting such as a university laboratory [108].

Regarding depressive symptoms and quality of life, we observed no differences between OR trials and CSR trials for either comparator group.

The difference in results we found between OR and CSR trials for studies with WLC comparators is in line with a previous meta-analysis on effectiveness of face-to-face CBT for anxiety disorders by Stewart and Chambless [9]. The small but significant effect size (*d*=–0.08; *P*<.05) they found indicated smaller improvements in more clinically representative patient studies than in less clinically representative studies.

Our findings are partly in keeping with Andersson and Hedman’s review on the effectiveness of iCBT for anxiety [21]. Results of that review suggested that effectiveness studies obtain similar effects to efficacy trials. Considering they only included studies comparing iCBT with face-to-face CBT, that conclusion corresponds to our results for iCBT compared with face-to-face CBT. However, it needs to be noted that Andersson and Hedman based their distinction between efficacy and effectiveness on the setting in which iCBT was delivered and not on recruitment strategy.

### Strengths and Limitations

A major strength of this study is that it is, to our knowledge, the first meta-analysis of iCBT for anxiety disorders comparing treatment outcomes between OR and CSR for both WLC and face-to-face CBT comparators. Furthermore, the studies comparing iCBT versus face-to-face CBT were head-to-head comparisons, generating direct evidence.

Some limitations in this study warrant caution in interpretation. First, the number of trials was relatively low for studies with face-to-face CBT comparators. Hence, finding no difference may have been caused by underpowered analyses.

Second, clinical representativeness of studies is often rated based on a multitude of criteria, besides recruitment type, such as setting of treatment delivery, experience of therapists, and flexibility in treatment manuals [9,109]. This means that the differences we found may have been caused by predictors not assessed in this study. In future research, considering multiple criteria of efficacy and effectiveness would be helpful to more thoroughly determine clinical representativeness of the studies and the association between clinical representativeness and treatment outcomes.

Third, in 3 included trials a mixed recruitment strategy was applied. This contamination may have led to some bias. Any such bias will have decreased the difference between OR and CSR trials found in the meta-analysis. We decided to include these trials because they reported clearly on their recruitment method and also the portion of participants recruited through an OR method versus a CSR method.

Fourth, when interpreting the subgroup analyses and metaregression analyses, it is important to bear in mind that the results were only observational. Direct comparisons are required to verify the findings presented here.

### Conclusions

This meta-analysis indicated that the effects of iCBT for anxiety disorders compared with WLC in CSR trials were smaller than effects found in OR trials. Hence, for patients with anxiety disorders in routine care, the impact of iCBT may not be as positive as for self-referred study participants recruited from the community. The difference between OR and CSR might be partly caused by a greater treatment adherence of self-referred patients and stricter exclusion criteria for severe depressive symptoms in studies with an OR method. A future challenge is to build a more robust body of evidence supporting the effectiveness of iCBT for anxiety disorders in routine care populations.

## Appendix

Multimedia Appendix 1 Search string.

Multimedia Appendix 2 Order of rating scales.

Multimedia Appendix 3 Characteristics of included studies.

Multimedia Appendix 4 Risk-of-bias assessment.

Multimedia Appendix 5 Secondary outcomes.

Multimedia Appendix 6 Funnel plot publication bias.

Multimedia Appendix 7 Metaregression and subgroup analyses.

## Abbreviations

BAI: Beck Anxiety Inventory
CBT: cognitive behavioral therapy
CSR: clinical service recruitment
DSM: Diagnostic and Statistical Manual of Mental Disorders
iCBT: internet-delivered cognitive behavioral therapy
NNT: number needed to treat
OR: open recruitment
PRISMA: Preferred Reporting Items for Systematic Reviews and Meta-Analyses
WLC: waitlist control
