# Supplementary material for: Internet-Delivered Cognitive Behavioral Therapy for Anxiety Disorders in Open Community Versus Clinical Service Recruitment: Meta-Analysis
Source: J Med Internet Res. 2019 Apr 17;21(4):e11706. doi: 10.2196/11706 (PMC6492068; doi:10.2196/11706)
Supplement: Multimedia Appendix 1 [file jmir_v21i4e11706_app1.pdf]

Multimedia Appendix 1. Search string

"Anxiety Disorders"[Mesh] OR anxiety disorder* [tiab] OR generalized anxiety disorder* [tiab] OR generalised anxiety disorder* [tiab] OR GAS [tiab] OR anxiety state* [tiab] OR agoraphobi* [tiab] OR panic* [tiab] OR phobi* [tiab] OR obsessive-compulsive [tiab] OR OCD [tiab] OR post-traumatic* [tiab] OR posttraumatic* [tiab] OR traumatic* [tiab] OR acrophobi* [tiab] OR claustrophobi* [tiab] OR ophidiophobi* [tiab] OR acute stress disorder* [tiab] OR castration anxiet* [tiab] OR death anxiet* [tiab]

AND

"Telemedicine"[Mesh] OR econsult*[tiab] OR e-consult*[tiab] OR eHealth*[tiab] OR e-health*[tiab] OR mhealth*[tiab] OR m-health*[tiab] OR mobile health*[tiab] OR remote consult*[tiab] OR Teleconsult*[tiab] OR Tele-consult*[tiab] OR telehealth*[tiab] OR tele-health*[tiab] OR telemedicin*[tiab] OR tele-medicin*[tiab] OR telemonitor*[tiab] OR tele-monitor*[tiab] OR blended [tiab] OR blending [tiab] OR web-based [tiab] OR webbased [tiab] OR online intervention* [tiab] OR online therap* [tiab] OR “Mobile applications” [Mesh] OR internet-based [tiab] OR web intervention* [tiab] OR mobile application* [tiab] OR tablet based [tiab] OR computerised [tiab] OR computerized [tiab] OR internet delivered [tiab] OR computer delivered [tiab] OR internet treatment* [tiab] OR internet cbt [tiab] OR computer augmented [tiab] OR computer assisted therap* [tiab]

AND

Randomized controlled trial[pt] OR controlled clinical trial[pt] OR randomized [tiab] OR placebo* [tiab] OR randomly[tiab] OR RCT [tiab] OR controlled trial* [tiab] OR clinical trial* [tiab] OR randomised [tiab]
