# Supplementary material for: Internet-Delivered Cognitive Behavioral Therapy for Anxiety Disorders in Open Community Versus Clinical Service Recruitment: Meta-Analysis
Source: J Med Internet Res. 2019 Apr 17;21(4):e11706. doi: 10.2196/11706 (PMC6492068; doi:10.2196/11706)
Supplement: Multimedia Appendix 2 [file jmir_v21i4e11706_app2.pdf]

# Multimedia Appendix 2. Order of rating scales

| Specific anxiety symptoms rating scale | Depression rating scales | Quality of Life (QOL) rating scales |
|---|---|---|
| ***Social anxiety disorder***<br>- Liebowitz Social Anxiety Scale (LSAS) [39,40]<br>- Social Phobia Scale (SPS) [41]<br>- Social Interaction Anxiety Scale (SIAS) [41]<br>- Social Phobia Screening Questionnaire (SPSQ) [42]<br>- Brief version of the Fear of Negative Evaluation Scale (BFNE) [43] | - Beck Depression Inventory (BDI) [35]<br>- Montgomery-Asberg Rating Scale (MADRS-S) [88]<br>- Patient Health Questionnaire-9 item scale (PHQ-9) [89]<br>- Centre of Epidemiological Studies-Depression scale (CES-D) [90]<br>- Depression Anxiety Stress Scales (DASS) [36] | - Quality Of Life Inventory (QOLI) [91]<br>- WHO Quality of Life Questionnaire (QOL) [92]<br>- EuroQol visual analogue scale (EQ-VAS) [93]<br>- Short-Form Health Survey-12 (SF-12) [94] |
| ***Generalized anxiety disorder***<br>- Generalized Anxiety Disorder -7 item scale (GAD-7) [44]<br>- Penn State Worry Questionnaire (PSWQ) [45]. | | |
| ***Panic disorder***<br>- Panic Disorder Severity Scale (PDSS) [46]<br>- Body Sensation Questionnaire (BSQ) [47]<br>- Panic and Agoraphobia Scale (PAS) [48] | | |
| ***Phobia***<br>- Fear Questionnaire (FQ) [49] | | |
