# Supplementary material for: Internet-Delivered Cognitive Behavioral Therapy for Anxiety Disorders in Open Community Versus Clinical Service Recruitment: Meta-Analysis
Source: J Med Internet Res. 2019 Apr 17;21(4):e11706. doi: 10.2196/11706 (PMC6492068; doi:10.2196/11706)
Supplement: Multimedia Appendix 3 [file jmir_v21i4e11706_app3.pdf]

Multimedia Appendix 3. Characteristics of included studies

Open recruitment studies

	**Study & year**	**Diagnosis & % comorbidity**	**N total**	**Age & gender (% women)**	**% Employed (total/ fulltime)**	**Education (% college degree**	**iCBT (no. of sessions, duration, support)**	**Control**	**Treatment dose (no. completed sessions) & Treatment adherence**	**Primary outcome measure + baseline score (mean, SD)<br>Secondary outcome measures**	**Inclusion criteria regarding 1) psychoactive medication & 2) comorbidity**
1.	Andersson et al., 2006 [58]	SAD<br>12.5%	64	37.3<br>51.6%	71.9% total<br>65.5% ft	43,8%	9 online sessions & 2 group sessions, 9 weeks, clinical support	WLC: delayed treatment, 9 weeks	7.5 sessions<br>83,3%	• BAI<br>iCBT: 13.6 (7.3)<br>WLC: 13.2 (6.6)<br>• MADRS-S<br>• QOLI	1) stable dose 3 months<br>2) no psychosis or substance abuse; score < 31 on the MADRS-S
2.	Andersson et al., 2009 [55]	Spider phobia	27	25.6<br>84.8%		26%	5 online sessions, 4 weeks, clinical support	f2f CBT: 2 sessions and maintenance program, clinical support, 2 weeks		• BAI<br>iCBT: 9.2 (5.4)<br>f2f CBT: 9.1 (5.3)<br>• BDI	1) stable dose 3 months<br>2) no other psychiatric problems requiring immediate treatment; no current depressive episode for >= 2 weeks in the last month
3.	Andersson et al., 2012a [56]	GAD	54	iCBT: 44.4 / 74.1%<br>WLC: 39.6 / 77.8%	61% total		8 online sessions, 8 weeks, clinical support	WLC: delayed treatment, 12 weeks	5.1 sessions<br>63.8%	• BAI<br>iCBT: 24.3 (9.10)<br>WLC: 23.7 (10.61)<br>• BDI<br>• QOLI	1) stable dose 6 weeks<br>2) not be severely depressed
4.	Andersson, et al., 2012b [59]	SAD	204	iCBT: 38.1 / 77.5%<br>WLC: 38.4 / 60%	74% ft	48%	9 online sessions, 9 weeks, clinical support	WLC: delayed treatment and access to discussion forum, 9 weeks	6.8 sessions<br>75.6%	• BAI<br>iCBT: 15.73 (7.98)<br>WLC: 16.47 (9.14)<br>• MADRS-S<br>• QOLI	1) stable dose 3 months<br>2) no other serious or dominant disorder (e.g. psychosis, substance misuse) that could influence outcome of the study; score <31 on the MADRS-S

5.	Andersson et al., 2013 [57]	Snake phobia	26	27.2<br>84.6%		27%	4 online sessions, 4 weeks, clinical support	f2f CBT: 2 sessions, clinical support, 2 weeks		• BAI<br>iCBT: 5.5 (3.7)<br>f2f CBT: 6.8 (3.3)<br>• BDI	2) no current depressive episode for >= 2 weeks in the last month
6.	Berger et al., 2009 [60]	SAD<br>26.9%	52	28.9<br>55.8%		42,3%	5 online sessions, 10 weeks, clinical support	WLC: delayed treatment, 10 weeks	4.3 sessions<br>85%	• LSAS<br>iCBT: 68.7 (16.9)<br>WLC: 75.0 (17.4)<br>• BDI	2) not currently meet diagnostic criteria for psychosis or borderline personality disorder
7.	Berger et al., 2014 [61]	SAD, GAD or PD(a)	132	35.1<br>56.1%	51.5% total<br>37.9% ft	59,8%	8 online sessions (tailored), 8 weeks, clinical support	WLC: delayed treatment, 8 weeks	7.3 sessions<br>90.6%	• BAI<br>iCBT: 33.8 (9.1)<br>WLC: 34.3 (8.4)<br>• BDI	1) stable dose for 3 months
8.							8 online sessions (disorder-specific), 8 weeks, clinical support		7.1 sessions<br>89.3%	iCBT: 34.3 (9.1)	
9.	Berger et al., 2017 [31]	SAD, GAD or PD(a)	139	42<br>70.5%	59.8% total<br>35.3% ft	52,5%	6 online sessions, 9 weeks, self-help	WLC: delayed treatment, access to treatment-as-usual, 9 weeks	3.9 sessions<br>65%	• BAI<br>iCBT: 34.9 (9.1)<br>WLC: 33.3 (10.3)<br>• BDI<br>• SF-12	1) stable dose 1 month
10.	Botella et al., 2010 [62]	SAD	127	24.4<br>79.2%		94,8%	Online sessions until feared situations are overcome, 2 months, self-help	f2f CBT; session once or twice a week, two months, clinical support		• BFNE<br>iCBT: 42.80 (9.32)<br>f2f CBT: 44.14 (8.97)<br>• BDI	2) not have a primary diagnosis of major depression; not be diagnosed for substance abuse or dependence, psychosis, or mental retardation
11.								WLC: delayed treatment, 4 weeks		WLC: 44.42 (9.99)	
12.	Carlbring et al., 2001 [63]	PD	41	34<br>70,7%			6 online sessions, 7-12 weeks, clinical support	WLC: delayed treatment, 7-12 weeks		• BAI<br>iCBT: 19.3 (6.2)<br>WLC: 21.5 (10.0)<br>• BDI<br>• QOLI	1) stable dose for 3 months<br>2) not suffer from any other psychiatric disorder in immediate need of treatment; score <21 on MADRS

13.	Carlbring et al., 2005<br>[64]	PD	49	35.0<br>71%			10 online sessions, 10 weeks, clinical support	f2f CBT: 10 sessions, 10 weeks, clinical support	7.4 sessions<br>74%	• BAI<br>iCBT: 18.7 (10.3)<br>f2f CBT: 24.5 (10.4)<br>• BDI<br>• QOLI	1) stable dose for 3 months<br>2) not suffer from any other psychiatric disorder in immediate need of treatment; score <21 on MADRS
14.	Carlbring et al., 2006<br>[65]	PD	60	36.7<br>60.0%		33%	10 online sessions, 10 weeks, clinical support	WLC: delayed treatment, 10 weeks	8.9 sessions<br>89%	• BAI<br>iCBT: 20.8 (10.0)<br>WLC: 19.5 (9.4)<br>• BDI<br>• QOLI	1) stable dose for 3 months<br>2) not suffer from any other psychiatric disorder in immediate need of treatment; score of <21 on MADRS
15.	Carlbring et al., 2007<br>[66]	SAD	57	iCBT: 32.4 / 58.6%<br>WLC: 32.9 / 71.4%		29.8%	9 online sessions, 9 weeks, clinical support	WLC: delayed treatment, 9 weeks		• BAI<br>iCBT: 14.5 (8.1)<br>WLC: 15.1 (8.8)<br>• MADRS-S<br>• QOLI	1) stable dose for 3 months<br>2) not currently meet diagnostic criteria for psychosis or substance misuse; score <31 on MADRS
16.	Carlbring et al., 2011<br>[67]	Anxiety disorder<br>50%	54	38.8<br>75.9%	56% total	41%	6-10 online sessions, 10 weeks, clinical support	WLC with attention control (online support group), 10 weeks	8 sessions<br>99.5%	• BAI<br>iCBT: 24.41 (9.97)<br>WLC: 21.00 (9.87)<br>• MADRS-S<br>• QOLI	1) stable dose for 3 months<br>2) score <31 on MADRS
17.	Christensen et al., 2014<br>[68]	GAD	21	iCBT: 25 / 75% WLC: 26 / 86%			10 online sessions, 7 f2f meetings with therapist/GP, 10 weeks, clinical support	WLC with attention control (website) and 7 f2f meetings with therapist/GP, 10 weeks		• GAD-7<br>iCBT: 11.5 (3.7)<br>WLC: 11.7 (4.8)<br>• CES-D	1) no prior treatment with Sertraline or monoamine oxidase inhibitors<br>2) no psychosis, bipolar disorder, primary diagnosis of depression
18.	Furmark et al., 2009<br>[69]	SAD	80	iCBT: 35 / 77.5%<br>WLC: 35.7 / 65.0%	52.5% ft		9 online sessions, 9 weeks, clinical support	WLC: delayed treatment, 9 weeks	6.4 sessions<br>71.2%	• LSAS<br>iCBT: 71.30 (22.4)<br>WLC: 71.28 (24.93)	1) stable dose 3 months<br>2) score <31 on MADRS
19.	Gallego et al., 2011<br>[52]	SAD	41	39.3<br>68.3%		43.9%	Online sessions until feared situations are overcome, 2 months, self-help	WLC: delayed treatment		• BFNE<br>iCBT: 39.41 (9.69)<br>WLC: 41.00 (9.72)	2) not suffering from alcohol or drug dependence or psychosis

20.	Johnston et al., 2011 [28]	GAD, SAD or PD(a) 69.7%	131	41.6 58.8%	44.3% ft		8 online sessions, 10 weeks, clinical support	WLC: delayed treatment, 10 weeks	7.1 sessions 88.6%	• GAD-7<br>iCBT: 11.63 (5.96)<br>WLC: 12.50 (4.80)<br>PHQ-9	1) no benzodiazepines, stable dose 3 months<br>2) no psychosis; no score >22 on the PHQ-9
21.							8 online sessions, 10 weeks, non-clinical support		7.6 sessions 94.6%	iCBT: 11.28 (5.18)	
22.	Kiropoulos et al., 2008 [70]	PD(a) 72.1%	86	39<br>iCBT: 71.7% f2f: 72.5%			6 online sessions, 12 weeks, clinical support	f2f CBT: 12 sessions, 12 weeks, clinical support		• DASS<br>iCBT: 3.67 (1.26)<br>f2f CBT: 3.68 (1.13)<br>• DASS<br>QOL	1) stable dose 12 weeks<br>2) no schizophrenia, alcohol or drug dependency, personality disorder
23.	Klein et al., 2006 [71]	PD(a) 89.1%	37	range 18-70 80.0%			6 online sessions, 6 weeks, clinical support	WLC: information, 6 weeks		• DASS<br>iCBT: 18.24 (8.5)<br>WLC: 16.13 (8.6)<br>• DASS	1) stable dose 4 weeks<br>2) no schizophrenia, alcohol or drug dependency, personality disorder
24.	Marks et al., 2004 [53]	PDa, SAD, specific phobia, agoraphobia	50	38 69%			9 steps in 6 blended sessions, 10 weeks, clinical support	f2f CBT; 6 sessions, 10 weeks, clinical support	4.2 sessions 70%	• FQ<br>iCBT: 6.1 (1.3)<br>f2f CBT: 6.7 (1.2)	1) no benzodiazepine or diazepam- equivalent dose of>5 mg/days; stable dose 4 weeks<br>2) no active psychotic illness
25.	Oromendia et al., 2016 [27]	PD(a)	77	40.7 68.8%			8 online sessions, 8 weeks, clinical support (non-scheduled)	WLC: delayed treatment, 8 weeks	3.5 session 44.3%	• BAI<br>iCBT: 34.92 (8.60)<br>WLC: 30.63 (9.53)<br>• BDI	1) stable dose 3 months<br>2) score =<25 on the BDI
26.							8 online sessions, 8 weeks, clinical support (scheduled)		5.5 sessions 68.3%	iCBT: 34.29 (10.97)	
27.	Paxling et al., 2011 [72]	GAD	89	39.3 79.8%			8 online sessions, 8 weeks, clinical support	WLC: delayed treatment, 8 weeks		• BAI<br>iCBT: 20.61 (10.64)<br>WLC: 20.98 (9.66)<br>• BDI<br>• QOLI	1) stable dose 1 month<br>2) score =<35 on MADRS-S, no severe mental illness such as active psychosis, alcohol abuse (as assessed by the AUDIT)
28.	Richards et al., 2006 [73]	PD(a)	32	36.6 68.6%			6 online sessions, 8 weeks, clinical support	WLC: information (online), 8 weeks		• DASS<br>iCBT: 19.58 (9.5)<br>WLC: 13.00 (7.4)<br>• DASS	1) stable dose 4 weeks<br>2) no schizophrenia, alcohol or drug dependency

										• QOLI	
29.							6 online sessions + 6 stress modules, 8 weeks, clinical support			iCBT: 21.09 (8.3)	
30.	Robinson et al., 2010 [74]	GAD	145	47<br>68.3%	43.4% ft		6 online sessions, 10 weeks, clinical support	WLC: delayed treatment, 11 weeks		• PSWQ<br>iCBT: 64.02 (9.27)<br>WLC: 65.81 (10.24)<br>• PHQ-9	1) stable dose 1 month<br>2) no psychotic mental illness (schizophrenia or bipolar disorder); no score >23 on the PHQ-9
31.							6 online sessions, 10 weeks, non-clinical support			iCBT: 63.12 (9.46)	
32.	Schulz et al., 2016 [75]	SAD<br>47.7%	149	35.4<br>53%	60.4% total<br>42.3% ft	42.3%	8 online sessions (individual), 12 weeks, clinical support	WLC: delayed treatment, 12 weeks	6.5 sessions<br>81.5%	• SPS<br>iCBT: 39.32 (11.64)<br>WLC: 37.35 (12.45)<br>• BDI<br>• SF-12	1) stable dose 1 month
33.							8 online sessions (group), 12 weeks, clinical support		6.4 sessions<br>79.4%	iCBT: 38.90 (14.04)	
34.	Titov et al., 2008a [76]	SAD	99	38.1<br>58.6%	55.6% ft		6 online sessions, 10 weeks, clinical support	WLC: delayed treatment, 10 weeks	5.2 sessions<br>86.7%	• SPS<br>iCBT: 34.02 (14.42)<br>WLC: 36.08 (16.63)<br>• PHQ-9<br>• WHODAS	1) stable dose 1 month<br>2) no psychotic mental illness, no score >19 on PHQ-9
35.	Titov et al., 2008b [77]	SAD	81	36.8<br>63%	54.3% ft		6 online sessions, 10 weeks, clinical support	WLC: delayed treatment, 10 weeks	5.5 sessions<br>91.7%	• SPS<br>iCBT: 34.15 (15.55)<br>WLC: 36.68 (14.62)<br>• PHQ-9<br>• WHODAS	1) stable dose 1 month<br>2) no psychotic mental illness, no score >19 on PHQ-9
36.	Titov et al., 2008c [78]	SAD	95	38<br>61%	58.1% ft		6 online sessions, 10 weeks, clinical support	WLC: delayed treatment, 10 weeks	5.4 sessions<br>89.8%	• SPS<br>iCBT: 34.71 (15.04)<br>WLC: 34.38 (18.77)<br>• PHQ-9	1) stable dose 1 month<br>2) no psychotic mental illness, no score >19 on PHQ-9

37.							6 online sessions, 10 weeks, self-help		4 sessions<br>66.2%	iCBT: 32.87 (17.02)	
38.	Titov et al., 2009<br>[79]	GAD	45	44<br>75.6%	40% ft		6 online sessions, 9 weeks, clinical support	WLC: delayed treatment, 9 weeks		• GAD-7<br>iCBT: 14.33 (4.50)<br>WLC: 13.62 (3.51)<br>• PHQ-9	1) stable dose 1 month<br>2) no psychotic mental illness, no score >23 on PHQ-9
39.	Titov et al., 2010 (GAD)<br>[30]	GAD<br>75.6%	34	39.5<br>67.9%	33.3% ft		6 online sessions, 8 weeks, clinical support	WLC: delayed treatment, 8 weeks		• PSWQ<br>iCBT: 66.17 (8.77)<br>WLC: 65.19 (9.78)	1) no benzodiazepines, stable dose 1 month<br>2) 2) no psychotic mental illness, no score >22 on PHQ-9
40.	Titov et al., 2010 (SAD)<br>[30]	SAD<br>75.6%	23	39.5<br>67.9%	33.3%ft		6 online sessions, 8 weeks, clinical support	WLC: delayed treatment, 8 weeks		• SPSQ<br>iCBT: 20.0 (9.49)<br>WLC: 18.45 (9.34)	1) no benzodiazepines, stable dose 1 month<br>2) no psychotic mental illness, no score >23 on PHQ-9
41.	Titov et al., 2010 (PD)<br>[30]	PD<br>75.6%	21	39.5<br>67.9%	33.3%ft		6 online sessions, 8 weeks, clinical support	WLC: delayed treatment, 8 weeks		• PDSS<br>iCBT: 12.80 (6.55)<br>WLC: 15.18 (5.67)	1) no benzodiazepines, stable dose 1 month<br>2) no psychotic mental illness, no score >23 on PHQ-9
42.	Titov et al., 2011<br>[29]	GAD, SAD or PD(a)<br>81%	36	43.9 73.0% (total sample	35% ft		8 online sessions, 10 weeks, clinical support	WLC: delayed treatment, 10 weeks	7.4 sessions<br>91.9%	• DASS<br>iCBT: 60.63 (25.72)<br>WLC: 45.76 (17.77)<br>• PHQ-9	1) no benzodiazepines; stable dose 1 month<br>2) no psychotic mental illness, no score >23 on PHQ-9
43.	Wims et al., 2010 [80]	PDa	54	iCBT: 39.5 / 72.4%<br>WLC: 45.1 / 80%	53.7% ft		6 online sessions, 8 weeks, clinical support	WLC: delayed treatment, 8 weeks		• PDSS<br>iCBT: 17.10 (4.84)<br>WLC: 16.44 (4.63)<br>• PHQ-9	1) no benzodiazepines, stable dose 1 month<br>2) no psychotic mental illness, no score >23 on PHQ-9

Clinical service recruitment studies

44.	Andrews et al., 2011 [81]	SAD	37	31.9<br>40.5%			6 online sessions, 8 weeks, clinical support	f2f group CBT, 7 4hr- sessions, 7 weeks, clinical support		• SPS<br>iCBT: 43.81 (20.7)<br>f2f CBT: 40.93 (15.4)<br>• WHODAS	
45.	Bell et al., 2012 [82]	GAD, SAD or PD(a)<br>47%	83	iCBT: 33.6 / 72.5% WLC: 36.9 / 62.8%	59% total		4 (GAD) or 6 (PD or SAD) online sessions, 12 weeks,	WLC: remained on wait-list *	2.3 sessions<br>57%	• BAI<br>iCBT: 24.0 (SE: 1.91)<br>WLC: 18.3 (SE: 1.72)	1) stable dose 6 weeks

						guided by research assistant*			• BDI	2) no substance dependence, psychotic illness, bipolar I disorder
46.	Bergstrom et al., 2010 [51]	PD(a)	104	iCBT: 33.8 / 64.0%<br>f2f: 34.6 / 59.3%			10 online sessions, 10 weeks, clinical support	f2f group CBT, 10 2hr-sessions, 10 weeks, clinical support	6.7 sessions<br>67%	• ASI<br>iCBT: 32.5 (11.6)<br>f2f CBT: 33.2 (12.4)<br>• MADRS-S	1) stable dose 2 months<br>2) no severe depression
47.	Bruinsma et al., 2016 [83]	PD(a)<br>58,3%	36	iCBT: 36.6<br>f2f: 39.4<br>61.1%		27.8%	12 online sessions, 12 weeks, clinical support	f2f CBT, 12 sessions, 12 weeks		• PAS<br>iCBT: 20.28 (9.31)<br>f2f CBT: 19.06 (9.18)	1) stable dose 2 months
48.	Hedman et al., 2011 [84]	SAD	126	iCBT: 35.2 / 62.5% f2f: 35.5 / 66.1%	74% total		15 online sessions, 15 weeks, clinical support	f2f group CBT, 1 individual session and 14 group sessions, 15 weeks, clinical support	9.3 sessions<br>62.2%	• BAI<br>iCBT: 18.7 (10.9)<br>f2f CBT: 18.6 (10.8)<br>• MADRS-S<br>• QOLI	1) stable dose 2 months<br>2) no substance abuse; no score >20 on the MADRS; no personality disorders cluster A or B
49.	Kok et al., 2014 [50]	PDa, SAD, specific phobia, agoraphobia	212	34.6<br>61%		57.1%	5 online sessions, 5 weeks, clinical support*	WLC: remained on wait-list * control and received a self-help book	3 sessions<br>37.5%	• BAI<br>iCBT: 45.01 (13.78)<br>WLC: 44.57 (13.16)<br>• CES-D	1) stable for duration of the intervention<br>2) no psychotic disorders
50.	Mathiasen et al., 2016 [85]	GAD, PD(a)	66	iCBT: 32.3 / 63.9%<br>WLC: 29.2 / 60.0%			9 modules, 9 weeks (or longer), clinical support*	WLC: remained on wait-list *,	5.3 sessions<br>58.7%	• BAI<br>iCBT: 22.86 (10.41)<br>WLC: 22.23 (8.54)<br>• BDI<br>• EQ-VAS	2) no Axis II disorders other than cluster C, bipolar disorder or depressive psychotic features
51.	Nordgreen et al., 2016 (PD) [54]	PD(a)<br>62.4%	69	34.9 / 60.1%	57% total		f2f psychoeducation session, 10 online sessions (10 weeks) + 12 f2f sessions, clinical support	f2f CBT: 12 sessions, clinical support		• BSQ<br>iCBT: 2.89 (0.78)<br>f2f CBT: 2.88 (0.79)<br>• BDI	1) stable dose 3 months<br>2) no current psychosis, substance dependency
52.	Nordgreen et al., 2016 (SAD) [54]	SAD<br>62.4%	104	31 / 46.2%	57% total		f2f psychoeducation session, 9 online sessions (9 weeks) + 12 f2f sessions, clinical support	f2f CBT: 12 sessions, clinical support		• SPS<br>iCBT: 42.55 (15.61)<br>f2f CBT: 43.83 (15.65)<br>• BDI	1) stable dose 3 months<br>2) no current psychosis, substance dependency

53.	Nordgren et al., 2014 [7]	GAD, SAD, PD(a), anxiety NOS 58%	100	iCBT: 35 / 66.0% WLC: 36 / 60.0%	47% total	41%	7-10 online modules, 10 weeks, clinical support	WLC with attention control, 10 weeks	4.6 sessions 53.5%	• BAI<br>iCBT: 21.18 (SE: 1.37)<br>f2f CBT: 21.31 (SE: 1.37)<br>• MADRS-S<br>• QOLI	1) stable dose 12 weeks<br>2) no score >30 on the MADRS

SAD: Social Anxiety Disorder; GAD: Generalized Anxiety Disorder; PD: Panic Disorder; PD(a): Panic Disorders with agoraphobia; iCBT: Internet-based Cognitive Behavioural Therapy; f2f CBT: face-to-face Cognitive Behavioural Therapy; WLC: Wait List Control

* Participants in both groups were on a wait-list for intensive outpatient treatment
