# Supplementary material for: Internet-Delivered Cognitive Behavioral Therapy for Anxiety Disorders in Open Community Versus Clinical Service Recruitment: Meta-Analysis
Source: J Med Internet Res. 2019 Apr 17;21(4):e11706. doi: 10.2196/11706 (PMC6492068; doi:10.2196/11706)
Supplement: Multimedia Appendix 4 [file jmir_v21i4e11706_app4.pdf]

Multimedia Appendix 4. Risk of bias assessment

Risk of bias graph OR trials

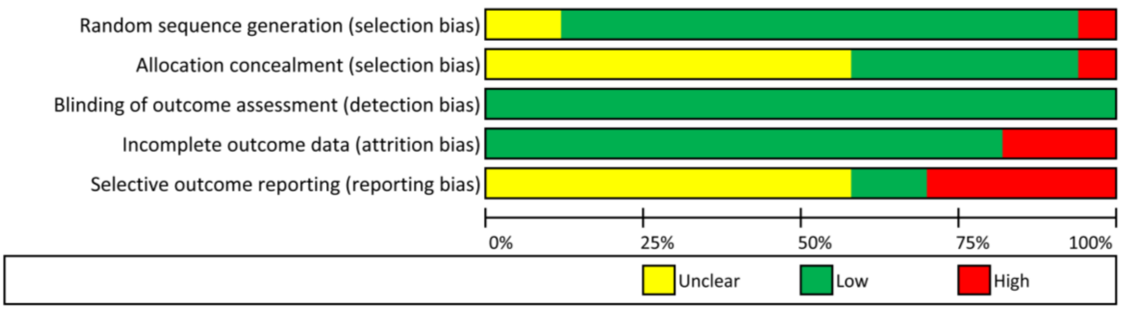

Risk of bias assessment OR trials per study

	sequence generation	allocation concealment	blinding outcome assessors	blinding participants / personnel	incomplete outcome data	selective outcome reporting
Andersson 2006	+	?	+	-	+	?
Andersson 2009	?	?	?	-	-	?
Andersson 2012a	+	+	+	-	+	+
Andersson 2012b	+	+	+	-	+	-
Andersson 2013	+	?	+	-	+	?
Berger 2009	+	?	+	-	+	?
Berger 2014	+	+	-	-	+	?
Berger 2017	+	+	+	-	+	-
Botella 2010	?	?	+	-	-	?
Carlbring 2001	?	?	+	-	+	?
Carlbring 2005	+	?	+	-	+	?
Carlbring 2006	+	?	+	-	+	?
Carlbring 2007	+	+	+	-	-	?
Carlbring 2011	+	+	+	-	+	?
Christensen 2014	+	?	+	-	+	?
Furmark 2009	+	+	+	-	+	?
Gallego 2011	-	?	?	-	-	?
Johnston 2011	+	+	+	-	+	-
Kiropoulos 2008	+	?	?	-	+	?
Klein 2006	-	-	-	-	+	?
Marks 2004	+	+	+	-	-	?
Oromendia 2016	+	+	+	-	+	+
Paxling 2011	+	+	+	-	+	?
Richards 2006	?	?	+	-	+	?
Robinson 2010	+	-	+	-	+	+
Schulz 2016	+	+	+	-	+	-
Titov 2008a	+	?	+	-	+	-
Titov 2008b	+	?	+	-	+	-
Titov 2008c	+	?	+	-	+	-
Titov 2009	+	?	+	-	-	+
Titov 2010	+	?	+	-	+	-
Titov 2011	+	?	+	-	+	-
Wims 2010	+	?	-	-	+	-

Risk of bias graph CSR trials

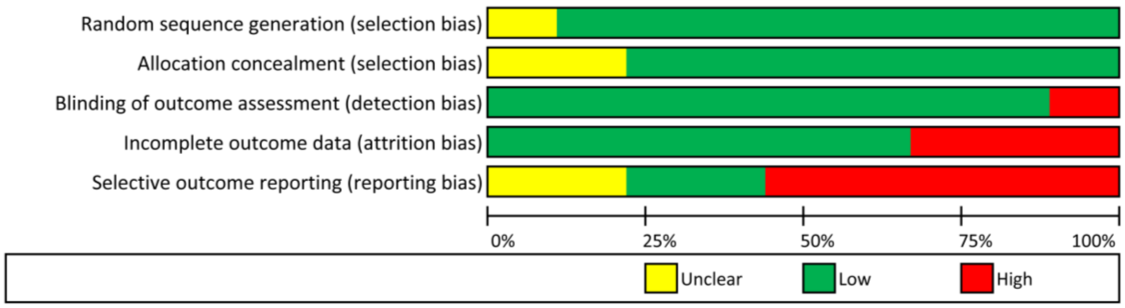

Risk of bias assessment CSR trials per study

	sequence generation	allocation concealment	blinding outcome assessors	blinding participants / personnel	incomplete outcome data	selective outcome reporting
Andrews 2011	?	?	+	-	+	-
Bell 2012	+	+	+	-	+	?
Bergström 2010	+	?	+	-	-	+
Bruinsma 2016	+	?	-	-	-	?
Hedman 2011	+	+	+	-	+	-
Kok 2014	+	+	+	-	-	-
Mathiasen 2016	+	+	+	-	+	-
Nordgreen 2016	+	+	-	-	+	-
Nordgren 2014	+	+	+	-	+	+
