# Supplementary material for: Internet-Delivered Cognitive Behavioral Therapy for Anxiety Disorders in Open Community Versus Clinical Service Recruitment: Meta-Analysis
Source: J Med Internet Res. 2019 Apr 17;21(4):e11706. doi: 10.2196/11706 (PMC6492068; doi:10.2196/11706)
Supplement: Multimedia Appendix 5 [file jmir_v21i4e11706_app5.pdf]

Multimedia Appendix 6. Secondary outcomes

Main effects of OR trials and CSR trials comparing iCBT to WLC and iCBT to f2f CBT at post-test, secondary outcomes

	n_{co}	n	g (95% CI)	*P-value*	I^2	NNT	Between-groups Q (*P-value*)
**Depressive symptoms**							
**WLC control**							
Open recruitment	32	2293	0.60 (0.51-0.68)	<.001	60	3	1.43 (.23)
Clinical recruitment	4	446	0.36 (0.17-0.54)	<.001	25	4	
**F2f CBT**							
Open recruitment	5	286	-0.05 (-0.29-0.18)	.65	34	-35	0.85 (.36)
Clinical recruitment	4	392	0.12 (-0.08-0.31)	.25	0	14	
**Quality of life**							
**WLC control**							
Open recruitment	15	1167	0.43 (0.31-0.55)	<.001	0	4	0.05 (.83)
Clinical recruitment	2	167	0.47 (0.16-0.77)	.003	80	3	
**F2f CBT**							
Open recruitment	2	298	0.09 (-0.24-0.43)	.59	0	19	0.48 (.49)
Clinical recruitment	2	163	0.25 (-0.06-0.56)	.11	0	7	

Note. WLC=wait list control; F2f CBT=face-to-face cognitive behavioural therapy; n_{comp}=number of comparisons; n=number of respondents;
