# Supplementary figures and images for: Internet-Delivered Cognitive Behavioral Therapy for Anxiety Disorders in Open Community Versus Clinical Service Recruitment: Meta-Analysis
Source: J Med Internet Res. 2019 Apr 17;21(4):e11706. doi: 10.2196/11706 (PMC6492068; doi:10.2196/11706)

## Multimedia Appendix 5. Funnel plot publication bias

iCBT compared to WLC

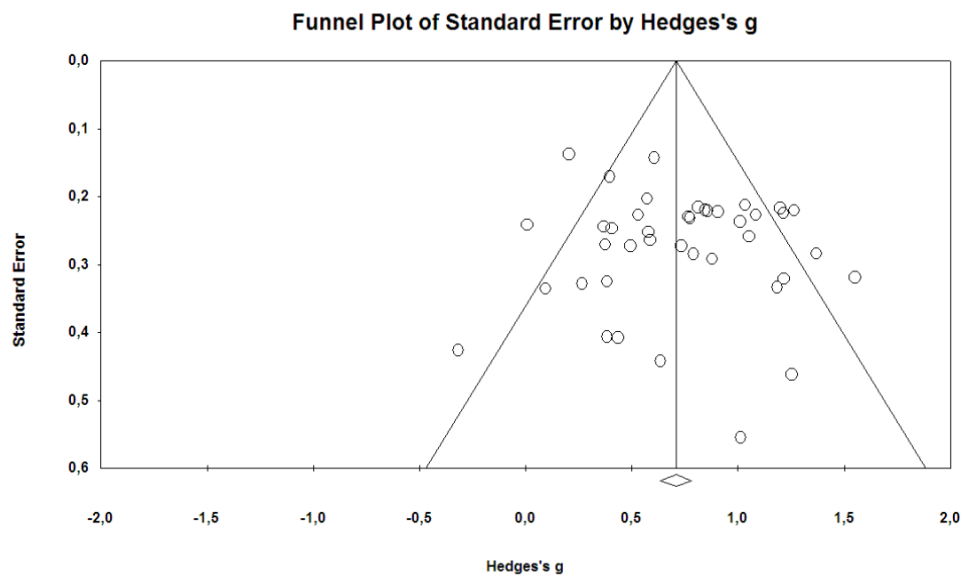

iCBT compared to f2f CBT

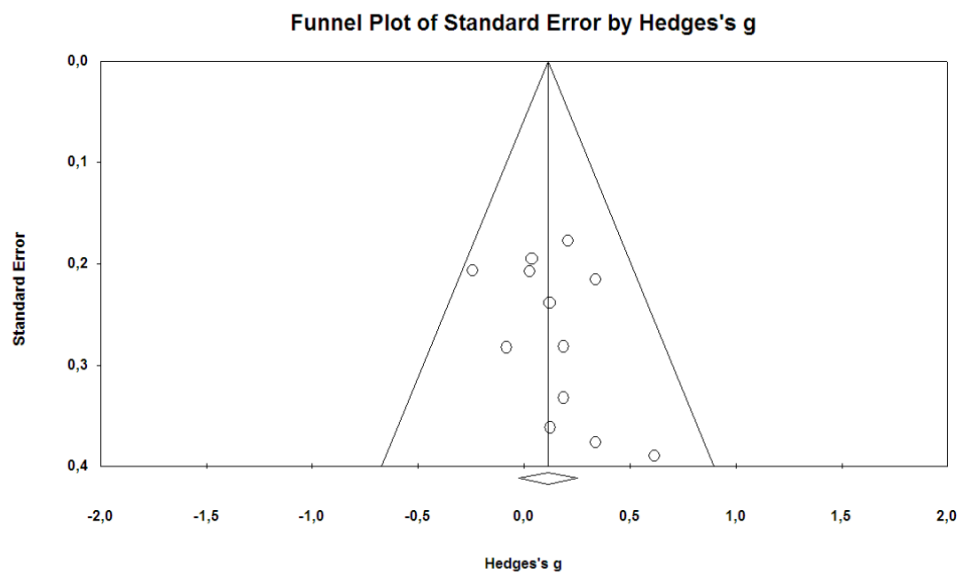

Supplement: Multimedia Appendix 6 [file jmir_v21i4e11706_app6.pdf]
