# Supplementary material for: Internet-Delivered Cognitive Behavioral Therapy for Anxiety Disorders in Open Community Versus Clinical Service Recruitment: Meta-Analysis
Source: J Med Internet Res. 2019 Apr 17;21(4):e11706. doi: 10.2196/11706 (PMC6492068; doi:10.2196/11706)
Supplement: Multimedia Appendix 7 [file jmir_v21i4e11706_app7.pdf]

Multimedia Appendix 7. Meta-regression and subgroup analyses

Results of meta regression analyses with sample characteristics

	**Association with effect size**
**Age**	WLC: slope=0.02, *P*=.14<br>F2f CBT: slope=0.00, *P*=.92
**Gender**	WLC: slope=-0.01, *P*=.22,<br>F2f CBT: slope=0.00, *P*=.37
**Baseline BAI score**	WLC: slope = -0.00, *P*=.69<br>F2f CBT: slope=-0.02, *P*=.35
**Baseline SPS score**	WLC: slope= 0.03, *P*=.60
**Treatment dose**	WLC: slope=0.10, *P* =.003<br>F2f CBT: slope=0.05, *P* =0.42
**Treatment adherence**	WLC: slope=0.01, *P* <.001<br>F2f CBT: slope=-0.00, *P* =.81

Results of subgroup analysis based on exclusion of severely depressed patients

	**N_{co}**	**g (95% CI)**	***P-value***	**I^2**	**NNT**	**Between-groups Q (*P-value*)**
**WLC**						
No exclusion of depressive patients	16	0.50 (0.39-0.62)	<.001	46	3	8.06 (.005)
Exclusion of depressive patients	25	0.84 (0.75-0.94)	<.001	47	2	
**F2f CBT**						
No exclusion of depressive patients	10	0.09 (-0.07-0.24)	.28	0	19	0.46 (.50)
Exclusion of depressive patients	2	0.20 (-0.09-0.50)	.18	0	8	
